# Tranexamic Acid Ameliorated Bleeding Tendency in Abdominal Aortic Aneurysm-Induced Chronic Disseminated Intravascular Coagulation

**DOI:** 10.7759/cureus.84822

**Published:** 2025-05-26

**Authors:** Seiichi Miwa

**Affiliations:** 1 Department of Internal Medicine, Tokoha University Rehabilitation Hospital, Hamamatsu, JPN

**Keywords:** abdominal aortic aneurysm, bleeding tendency, chronic disseminated intravascular coagulation, enhanced-fibrinolytic type, tranexamic acid

## Abstract

An 84-year-old man with a history of cerebral infarction, inoperable abdominal aortic aneurysm (AAA), and chronic kidney disease-related anemia was admitted for rehabilitation. He had experienced recurrent gastrointestinal bleeding before admission, initially attributed to dual antiplatelet therapy. After admission, progressive subcutaneous hemorrhage developed, prompting detailed coagulation-fibrinolysis testing, which confirmed chronic disseminated intravascular coagulation (DIC) (enhanced-fibrinolytic type) secondary to AAA. Given his advanced age and overall condition, oral tranexamic acid was initiated, resulting in hemorrhage improvement and stabilization of coagulation parameters. Retrospective evaluation suggested that prior gastrointestinal bleeding episodes were more likely attributable to chronic DIC. This case highlights the importance of considering chronic DIC in patients with recurrent, unexplained bleeding, even when they have been evaluated by multiple specialists, and suggests that individualized antifibrinolytic therapy, although not standard, may serve as a practical and familiar option for general internists in carefully selected inoperable elderly patients.

## Introduction

An aortic aneurysm is a vascular disorder characterized by pathological dilation of the aorta [1]. It is often asymptomatic and typically discovered during screening or incidentally through imaging modalities such as ultrasonography, computed tomography (CT), or magnetic resonance imaging (MRI) [1]. If left untreated, an aortic aneurysm may continue to enlarge and eventually rupture, leading to life-threatening catastrophic bleeding. Epidemiologically, the prevalence of aortic aneurysms increases with age [1]. In Japan, where life expectancy is high, elderly patients with aortic aneurysm are not uncommon [2].

One of the recognized complications of aortic aneurysm is chronic disseminated intravascular coagulation (DIC), which predominantly enhances fibrinolytic activity rather than coagulation [3,4]. This complication is also observed in aortic dissection, and both conditions can manifest as gingival, subcutaneous, nasal, intramuscular, joint, urinary tract, or gastrointestinal bleeding [5-16]. Because chronic fibrinolytic-type DIC associated with aortic aneurysms or aortic dissections is rare [4], its symptoms may be overlooked. This is partly due to the common perception of DIC as an acute condition, which can lead to underrecognition of its chronic, fibrinolytic forms. Lack of familiarity with this subtype may also contribute to delayed diagnosis. Moreover, even when diagnosed, its management remains challenging.

Tranexamic acid is an antifibrinolytic agent that inhibits plasminogen activation and prevents plasmin formation, thereby reducing fibrin degradation [4]. While tranexamic acid monotherapy is not generally recommended for chronic fibrinolytic-type DIC associated with aortic aneurysms [4], several reports have demonstrated its clinical utility [7,9,12,14,15]. Here, we report a patient with an inoperable aortic aneurysm and a history of two episodes of gastrointestinal bleeding who presented with subcutaneous hemorrhage. The patient was ultimately diagnosed with chronic fibrinolytic-type DIC and was successfully treated with oral tranexamic acid.

Despite being evaluated and managed by multiple specialists during previous events, the underlying condition remained undiagnosed. This case underscores the diagnostic challenges of recognizing chronic DIC in elderly patients, in whom symptoms may be subtle or misattributed, particularly when recurrent bleeding occurs without an obvious cause. It also suggests that tranexamic acid, although not standard therapy, may represent a practical and familiar option for general internists when used with appropriate clinical judgment.

## Case presentation

**Figure 1:**
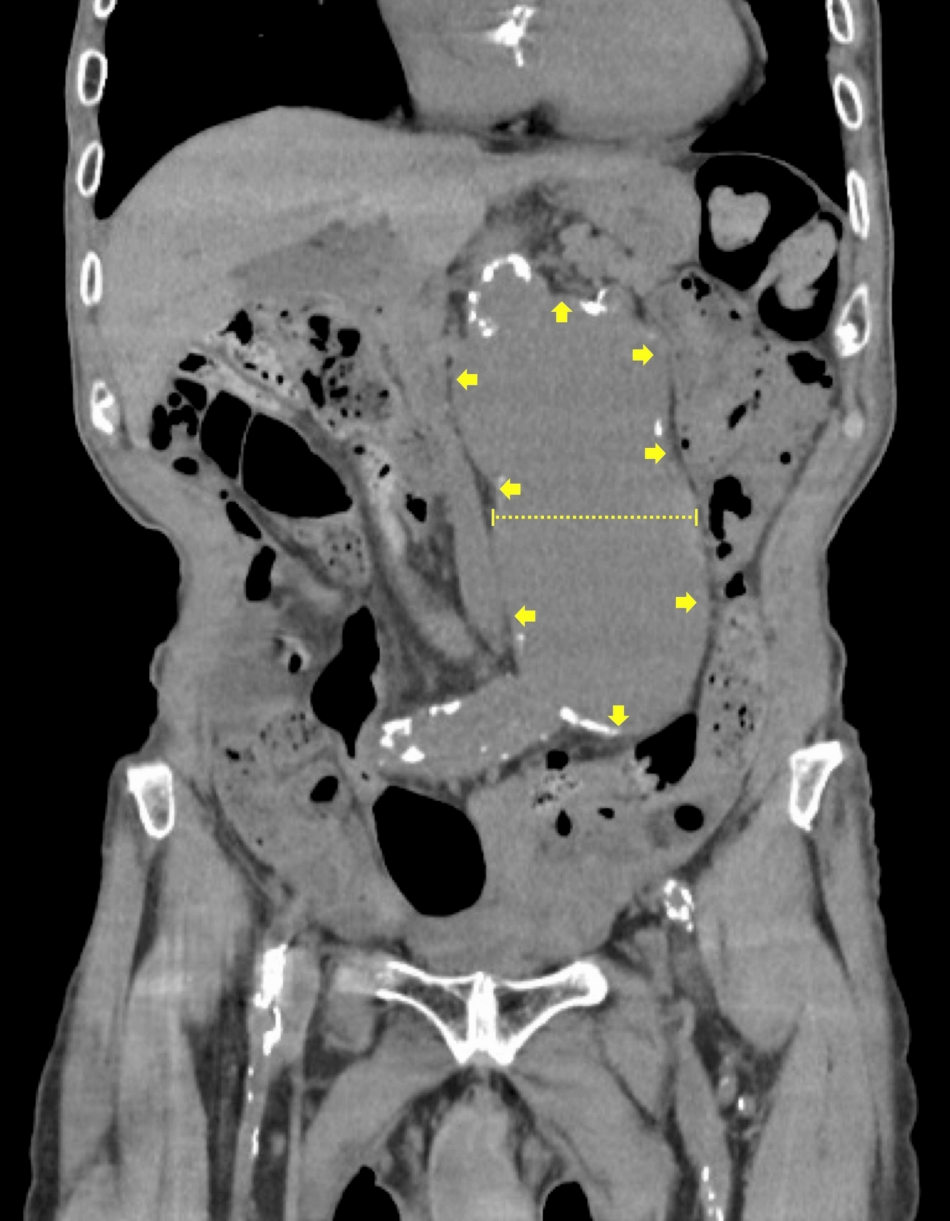
Abdominal CT showing a large aortic aneurysm. Coronal abdominal CT image taken 18 days prior to admission, demonstrating a large abdominal aortic aneurysm (arrows) with a diameter of 68.8 mm.

An 84-year-old man was admitted to the hospital for rehabilitation due to disuse syndrome. His medical history included cerebral infarction, an abdominal aortic aneurysm (AAA) (Figure 1), and anemia associated with chronic kidney disease (CKD). These conditions had been monitored at another institution. A cerebral infarction involving the left middle cerebral artery had been diagnosed seven years prior, and since then, he had been prescribed dual antiplatelet therapy (DAPT) with aspirin and clopidogrel by a neurologist due to recurrent strokes, left internal carotid artery occlusion, and coronary artery occlusion. AAA had been identified at the time of his cerebral infarction diagnosis, but was deemed inoperable due to high surgical risk. Based on mutual decision-making, an observation strategy was adopted, and the AAA was monitored two to three times per year with abdominal CT at another institution. His CKD-related anemia had been managed with erythropoiesis-stimulating agents (ESAs).

Over the past 12 weeks, he had been hospitalized twice at another institution for gastrointestinal bleeding. He experienced bleeding from gastric polyps, which was treated with endoscopic clipping and blood transfusions. Eighteen days before admission, he developed a hemorrhagic rectal ulcer, which was treated with endoscopic clipping, argon plasma coagulation, and blood transfusion. The attending gastroenterologists attributed both bleeding episodes to DAPT, with constipation also considered a contributing factor in the latter event.

On admission, his vital signs were as follows: body temperature of 36.7℃; blood pressure of 104/77 mm Hg; and heart rate of 54 beats per minute. He had right-sided paresis and was chair-bound as a result of prior cerebrovascular accidents. Laboratory findings revealed a white blood cell count (5030/μL) and platelet count (153,000/μL) within normal range, hemoglobin level of 10.6 g/dL, blood urea nitrogen level of 22.0 mg/dL, and creatinine level of 1.58 mg/dL.

**Figure 2:**
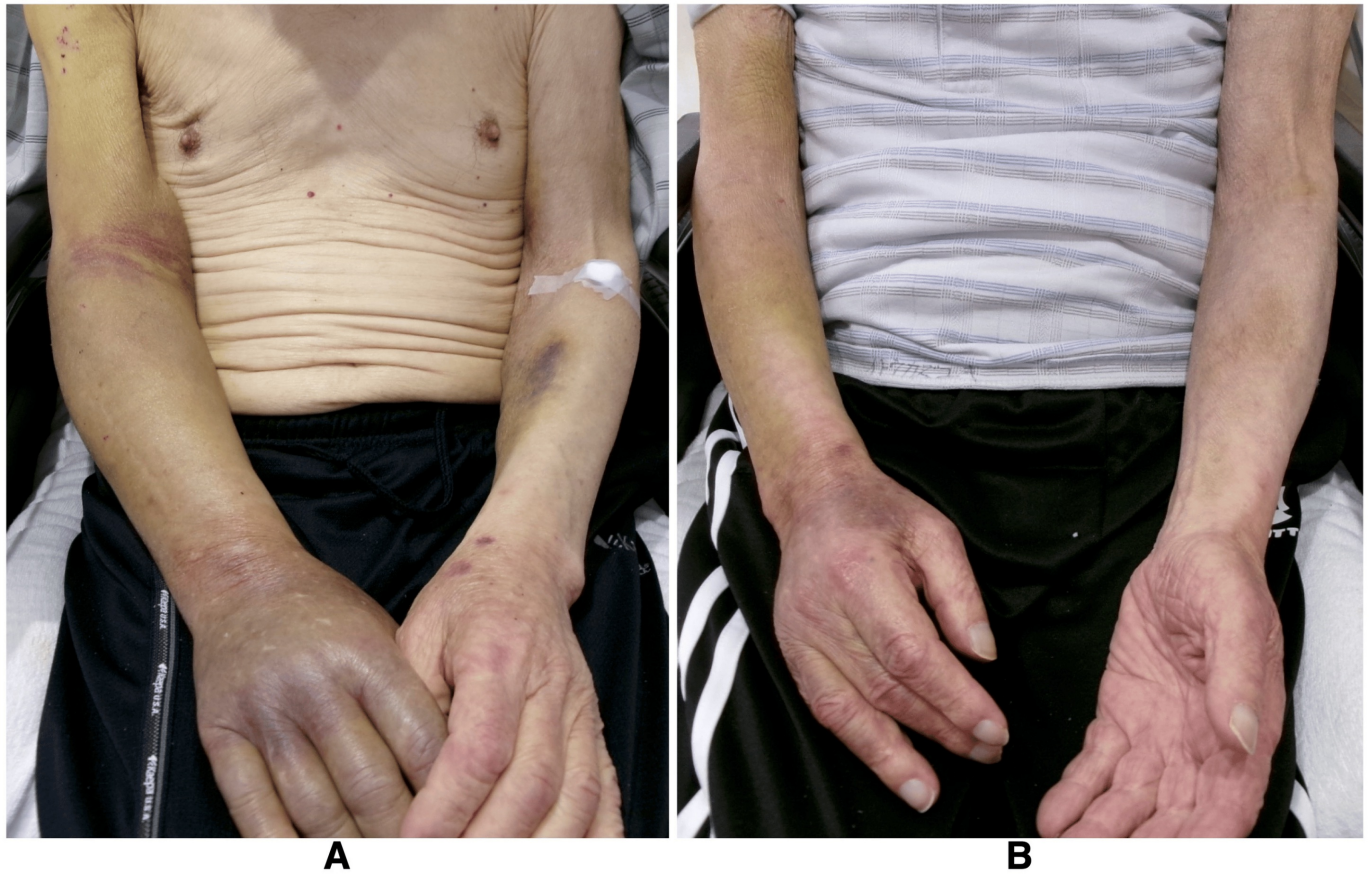
Subcutaneous hemorrhage associated with chronic fibrinolytic-type disseminated intravascular coagulation. (A) Subcutaneous hemorrhage extending from the right upper to the lower arm on hospital day 38. (B) Resolution of the hemorrhage following the initiation of oral tranexamic acid.

There were no significant events during the initial period after admission. DAPT with aspirin and clopidogrel, originally initiated for secondary stroke prevention, was continued. On hospital day 17, subcutaneous hemorrhage was observed in the left upper arm. Given the possibility of DAPT-related side effects, the condition was initially monitored. No pharmacological prophylaxis for deep vein thrombosis, such as heparin, was administered. However, the hemorrhage did not improve and, by hospital day 38, had extended from the right upper to the lower arm (Figure 2A).

A detailed coagulation-fibrinolysis workup revealed a decreased platelet count (95,000/μL), an elevated D-dimer level (43.2 μg/mL; reference value: <1), and a low fibrinogen level (72 mg/dL; reference value: 170-410). The international normalized ratio of prothrombin time (PT-INR) was 1.16 (reference value: 0.9-1.13), and the activated partial thromboplastin time (APTT) was 39.8 seconds (reference value: 26.0-38.0) (Table 1).

**Table 1:** Laboratory data on hospital day 38. WBC: white blood cells; AST: aspartate aminotransferase; ALT: alanine aminotransferase; LD: lactate dehydrogenase; ALP: alkaline phosphatase; BUN: blood urea nitrogen; Cr: creatinine; Na: sodium; K: potassium; Cl: chloride; CRP: C-reactive protein; PT-INR: international normalized ratio of prothrombin time; APTT: activated partial thromboplastin time; FDP: fibrin degradation product.

Parameter	Value	Reference value
WBC count (/μL)	3190	3500-9700
Neutrophils (%)	69	42-74
Lymphocytes (%)	23	18-50
Eosinophils (%)	3	0-7
Hemoglobin (g/dL)	7.5	13.6-18.3
Platelet count (x10⁴/μL)	9.5	14.0-37.9
Total protein (g/dL)	6.1	6.5-8.2
Albumin (g/dL)	3.3	3.8-5.2
AST (U/L)	19	10-40
ALT (U/L)	26	5-45
LD (U/L)	195	120-245
ALP (U/L)	88	38-113
BUN (mg/dL)	24.9	8.0-20.0
Cr (mg/dL)	1.83	0.65-1.09
Na (mEq/L)	138	135-145
K (mEq/L)	4.4	3.5-5.0
Cl (mEq/L)	99	98-108
CRP (mg/dL)	<0.05	<0.3
PT-INR	1.16	0.9-1.13
APTT (s)	39.8	26.0-38.0
Fibrinogen (mg/dL)	72	170-410
D-dimer (μg/mL)	43.2	<1.0

**Figure 3:**
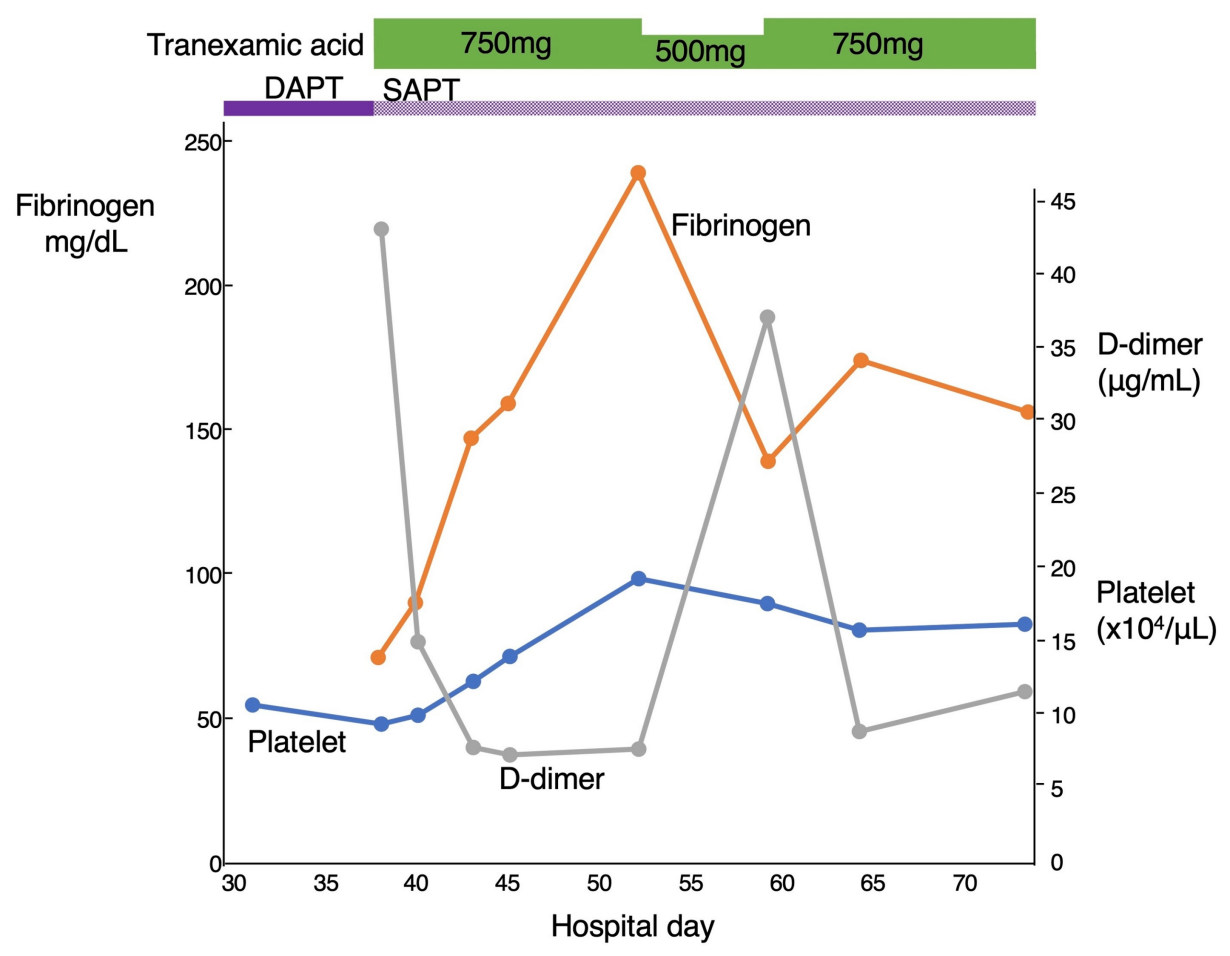
Clinical course, treatment, and changes in coagulation markers. Timeline of treatment interventions and corresponding changes in coagulation markers throughout the hospital stay. DAPT: dual antiplatelet therapy; SAPT: single antiplatelet therapy.

Possible causes such as malignancy and liver disease were excluded prior to admission at another hospital through chest and abdominal CT scans and endoscopy (no additional CT was performed at our hospital). In addition, infection was ruled out due to the absence of fever and elevated inflammatory markers at that time. Based on these findings, chronic DIC secondary to AAA was considered the most likely diagnosis. Given the patient's advanced age and general condition, oral tranexamic acid (750 mg per day) was initiated with informed consent, with aspirin continued as monotherapy after discontinuation of clopidogrel. Following the initiation of tranexamic acid, subcutaneous hemorrhage gradually improved (Figure 2B), and coagulation parameters remained stable, including a decrease in D-dimer (7.8 μg/mL) and increases in fibrinogen (240 mg/dL) and platelet count (194,000/μL) (Figure 3).

Two weeks after starting treatment, the dose was reduced to 500 mg per day due to clinical stabilization and in consideration of the patient’s CKD. However, this led to an increase in D-dimer (37.2 μg/mL) and a decrease in fibrinogen (140 mg/dL), suggesting reactivation of fibrinolysis and necessitating a return to the original dose of 750 mg per day. Furthermore, fibrin degradation product (FDP) was elevated at 22.0 μg/mL (reference value: <5 μg/mL), thrombin-antithrombin complex (TAT) was elevated at 36.3 ng/mL (reference value: <4.0), and plasmin-α2 plasmin inhibitor complex (PIC) was elevated at 6.8 μg/mL (reference value: <0.8), confirming the diagnosis of chronic fibrinolytic-type DIC according to the diagnostic criteria of the Japanese Society on Thrombosis and Hemostasis (Table 2) [3].

**Table 2:** Laboratory data on hospital day 64. WBC: white blood cells; BUN: blood urea nitrogen; Cr: creatinine; CRP: C-reactive protein; PT-INR: international normalized ratio of prothrombin time; APTT: activated partial thromboplastin time; FDP: fibrin degradation product; TAT: thrombin-antithrombin complex; PIC: plasmin-α2 plasmin inhibitor complex.

Parameter	Value	Reference value
WBC count (/μL)	3740	3500-9700
Hemoglobin (g/dL)	8.9	13.6-18.3
Platelet count (x10⁴/μL)	15.9	14.0-37.9
BUN (mg/dL)	22.8	8.0-20.0
Cr (mg/dL)	1.68	0.65-1.09
CRP (mg/dL)	0.11	<0.3
PT-INR	1.1	0.9-1.13
APTT (s)	33.6	26.0-38.0
Fibrinogen (mg/dL)	175	170-410
D-dimer (μg/mL)	9.0	<1.0
FDP (μg/mL)	22.0	<5.0
TAT (ng/mL)	36.3	<4.0
PIC (μg/mL)	6.8	<0.8

Retrospective evaluation suggested that the two prior episodes of gastrointestinal bleeding were more likely to be attributable to chronic DIC rather than DAPT (Table 3). It took over a month for his bleeding tendency to stabilize, and discharge was further delayed due to difficulty arranging placement in a care facility. On hospital day 80, the patient was discharged in stable condition. However, one month post discharge, he succumbed to an AAA rupture.

**Table 3:** Coagulation data before admission. PT-INR: international normalized ratio of prothrombin time; APTT: activated partial thromboplastin time; FDP: fibrin degradation product.

Time (before admission)		15 weeks	12 weeks	9 weeks	7 weeks	18 days	2 weeks
Clinical events			Gastric polyps bleeding			Rectal ulcer bleeding	Rectal bleeding
	Reference value						
Hemoglobin (g/dL)	13.6-18.3	9.7	6.1	10.3	7.4	8.9	6.9
Platelet count (×10⁴/μL)	14.0-37.9	16.0	11.4	13.6	11.4	12.7	11.1
PT-INR	0.9-1.13	0.99	1.18	1.05	0.96	-	-
APTT (s)	26.0-38.0	28.3	23.1	39.6	27.7	-	-
Fibrinogen (mg/dL)	170-410	218	125	139	165	-	-
D-dimer (μg/mL)	<1.0	46.8	-	42.3	61.6	-	-
FDP (μg/mL)	<5.0	69.0	-	-	-	-	-

## Discussion

DIC is a complex, systemic disorder characterized by widespread activation of the coagulation cascade, resulting in both thrombotic and hemorrhagic complications [3]. It is not uncommon in hospitalized patients, with reported incidences of approximately 1% among those in intensive care units [17]. DIC is classified into three types: the suppressed-fibrinolytic type (thrombosis-dominant), the balanced-fibrinolytic type (mild thrombosis and bleeding), and the enhanced-fibrinolytic type (bleeding-dominant) [3,4]. The suppressed type is commonly associated with sepsis, the balanced type with solid tumors, and the enhanced type with vascular abnormalities, including aortic aneurysm or aortic dissection, and acute promyelocytic leukemia (APL) [3,4]. DIC secondary to sepsis is generally considered an acute form, whereas DIC associated with malignancies or vascular abnormalities tends to follow a chronic course. The rarity of chronic DIC caused by aortic aneurysm, along with compensatory homeostatic mechanisms, can obscure clinical symptoms and delay diagnosis. In fact, elevated FDP and D-dimer levels have been reported in up to 40% of such cases, even when overt bleeding is absent [4,18]. In this case, the diagnosis was established when subcutaneous hemorrhage appeared; however, two previous episodes of gastrointestinal bleeding were also likely manifestations of chronic DIC.

The management of DIC primarily focuses on addressing the underlying cause. In cases of chronic DIC associated with an aortic aneurysm or aortic dissection, the most effective approach is surgical repair of the aorta [10,13], as it eliminates the source of ongoing coagulation activation. However, surgical intervention is often not feasible, particularly in those who have advanced age and comorbidities, as observed in the present case. For patients who are not candidates for surgery, the management of chronic DIC secondary to aortic disease remains challenging. Previous reports have suggested anticoagulant therapy [6,8,16,19], antifibrinolytic therapy [7,9,12,14,15], or a combination of both [5,11] as potential treatment options. Anticoagulant therapies include heparins, synthetic protease inhibitors such as nafamostat mesylate, and direct oral anticoagulants (DOACs), whereas antifibrinolytic therapy consists of tranexamic acid [4]. Theoretically, anticoagulation is recommended for chronic DIC in the context of an aortic aneurysm to prevent thrombotic complications [4]. However, in clinical practice, the presence of active bleeding often leads to reluctance in initiating anticoagulation, as it necessitates careful assessment of the risk of exacerbating hemorrhage [18]. In this case, given the patient's condition, oral tranexamic acid was administered, resulting in an improvement in both subcutaneous hemorrhage and coagulation-fibrinolysis parameters.

Enhanced-fibrinolytic DIC associated with aortic aneurysm is a rare and often overlooked condition, with an incidence ranging from 0.5% to 4.0% among patients with aortic aneurysms [4]. Predicting its occurrence requires a combination of clinical awareness, laboratory markers, and imaging findings. Among these, monitoring coagulation markers is the most practical approach for both early detection and ongoing assessment. In particular, significantly reduced fibrinogen levels, markedly elevated D-dimer and FDP levels, and increased PIC levels are recognized as key indicators of enhanced-fibrinolytic DIC [3,4]. Additionally, an elevated FDP-to-D-dimer ratio serves as an important marker for suspecting this condition (Table 4) [4].

**Table 4:** Key features and diagnostic markers of chronic fibrinolytic-type DIC associated with aortic aneurysms. DIC: disseminated intravascular coagulation; FDP: fibrin degradation product; TAT: thrombin-antithrombin complex; PIC: plasmin-α2 plasmin inhibitor complex.

Red flag symptoms	Critical laboratory markers
• Recurrent unexplained bleeding (e.g., gastrointestinal and subcutaneous)	• Platelet count: decreased
• Bleeding in the absence of anticoagulants or trauma	• Fibrinogen: markedly decreased
• History of aortic aneurysm or dissection	• D-dimer: markedly elevated
	• FDP: markedly elevated
	• FDP/D-dimer ratio: increased 2-5
	• TAT: markedly elevated
	• PIC: markedly elevated

In the present case, FDP could not be assessed at the time of peak symptom severity along with D-dimer, which is a limitation. However, for reference, when the patient's condition showed signs of improvement, the FDP level was 22.0 μg/mL. Retrospective analysis of coagulation data in another hospital, 15 weeks prior to admission (three weeks before two episodes of gastrointestinal bleeding), revealed markedly elevated FDP (69.0 μg/mL) and D-dimer (46.8 μg/mL) levels (Table 3). These findings suggest that coagulation abnormalities were likely involved in the development of gastrointestinal bleeding, highlighting the potential role of coagulation marker monitoring in predicting disease progression and complications.

## Conclusions

Chronic fibrinolytic-type DIC associated with aortic aneurysms is rare. However, given the relative frequency of aortic aneurysms, clinicians should remain vigilant for this condition. Even when diagnosed, its management remains challenging, particularly in elderly patients with inoperable aortic aneurysms. The risk of rupture persists in these patients, underscoring the importance of careful monitoring and shared decision-making. Although not a standard treatment, oral tranexamic acid may serve as a practical alternative therapeutic option for controlling bleeding in selected cases. Early evaluation of coagulation and fibrinolytic markers may facilitate timely recognition and management of this condition.
